# Ninety-Day Oral Toxicity Assessment of an Alternative Biopolymer for Controlled Release Drug Delivery Systems Obtained from Cassava Starch Acetate

**DOI:** 10.1155/2015/390416

**Published:** 2015-09-14

**Authors:** Douglas Rossi Jesus, Lorena Neris Barbosa, Thiago Bruno Lima Prando, Leonardo Franco Martins, Francielli Gasparotto, Karla Moraes Rocha Guedes, Douglas Cardoso Dragunski, Emerson Luiz Botelho Lourenço, Paulo Roberto Dalsenter, Arquimedes Gasparotto Junior

**Affiliations:** ^1^Laboratory of Toxicology and Pharmacology of Natural Products, Paranaense University, P.O. Box 224, 87502-210 Umuarama, PR, Brazil; ^2^Department of Pharmacology, Federal University of Paraná, P.O. Box 19031, 81531-990 Curitiba, PR, Brazil; ^3^University Center Cesumar, P.O. Box 447, 87050-900 Maringá, PR, Brazil; ^4^Laboratory of Cardiovascular Pharmacology, Faculty of Health Sciences, Federal University of Grande Dourados, Rodovia Dourados-Itahum, km 12, P.O. Box 533, 79.804-970 Dourados, MS, Brazil

## Abstract

The large consumption of biodegradable films from cassava starch acetate (FCSA) as ingredients in food and pharmaceutical products requires the assessment of the possible toxicity of these products. The aim of this study was to investigate the toxicity of biodegradable film from cassava starch acetate after oral exposure of Wistar rats for 90 days. The amount of food consumed and the body weight were weekly monitored. Blood and urine samples were obtained for the assessment of serum parameters and renal function. Histopathological analyses in target organs were also performed. No evidence of clinical toxicity in hematological, biochemical, or renal parameters in the FCSA-treated animals was found. In addition, relative organ weight and histopathological evaluations did not differ between groups treated with FCSA and control. Data obtained suggest that the subchronic exposure to FCSA does not cause obvious signs of toxicity in Wistar rats, indicating possible safety of this biofilm.

## 1. Introduction

In recent decades, a new generation of biomaterials was introduced as alternative to reduce the degradation of perishable foods. In addition, pharmaceutical industries have applied edible films as adjuvants in controlled drug release systems [1–3]. Edible films are usually produced from natural and plentiful renewable sources, such as starches, lipids, proteins, or combinations of these compounds. Since the 1970s, starch has been considered a good candidate for the production of biodegradable films, consisting of low-cost renewable resources, including polysaccharides, amylose, and amylopectin [4]. Among other advantages, they are transparent, flavorless, tasteless, and colorless; moreover, several studies have shown their high potential in reducing lipid oxidation in fresh foods or as a carrier system for controlled delivery of drugs for various therapeutic applications [1, 2, 5].

Cassava (*Manihot esculenta* Crantz) is a major source of starch in Brazil. The species is distinguished by the large amount of amylose (~20%), a fact that significantly contributes to the preparation of new biomaterials [4]. When used to obtain polymeric materials, cassava starch has shown high fragility. Thus, it is common to add plasticizers such as glycerol, water, or sorbitol [6]. This procedure increases flexibility; however, starch becomes weaker and has increased permeability. Considering these apparent difficulties, starch acetylation is an important technique applied to native starches to provide thickness, strength, and reduced permeability. Moreover, as starch acetylation considerably decreases swelling and enzymatic degradation by intestinal amylase, this technique allows producing a highly effective film to be used in food and pharmaceutical industries [2, 3].

Although these edible films are actually very promising, the risks of prolonged use of these chemically modified natural compounds remain unknown. Few toxicological studies have been conducted with modified starches, and some data suggest higher prevalence of structural changes in the kidneys and intestines of mice after prolonged use [7].

Given the importance of these biopolymers and their possible use on a large scale, subchronic toxicity studies are essential steps in obtaining information on the safe use of these compounds. Thus, the aim of this study was to investigate the subchronic toxicity of biodegradable film obtained from cassava starch acetate after oral exposure of 60-day-old Wistar rats.

## 2. Materials and Methods

### 2.1. Materials

High-amylose cassava starch (AVEBE starches, Brazil) was used. Acetic anhydride (≥99%) and D-sorbitol (≥99%) were obtained from Sigma-Aldrich Co. (St. Louis, MO, USA) and Fluka Chemical Co.

### 2.2. Starch Acetate (SA) Synthesis and Preparation of Homogeneous Film from Cassava Starch Acetate (FCSA)

Acetylated starch was prepared from high-amylose cassava starch according to methodology previously described by Xie et al. [8]. 40% (w/v) high-amylose cassava starch slurry was placed into a reactor equipped with magnetic stirrer at 80°C. After pH adjustment to 8.0, acetic anhydride was added, and pH was maintained constant with NaOH solution. After 2 h, the mixture was neutralized by 0.5 M hydrochloric acid. The mixture was filtered and washed with excess distilled water until acetate ion was not detected by titration methods. The solid was dried at 45°C in vacuum oven and then sieved. To obtain FCSA, 3 g of SA was solubilized in 100 mL of distilled water at 85°C for 30 min under constant stirring. The solution obtained was added of sorbitol (10 wt.%) and spread on a glass plate coated with polyvinyl chloride. Films were dried for 5 hours in a ventilated oven (Binder KBF 240, Odil) under controlled temperature and humidity (40°C and 30%). Before experiments, FCSA was comminuted and solubilized in distilled water to facilitate administration.

### 2.3. Toxicological Studies

#### 2.3.1. Animals

Forty male and 40 female Wistar rats of similar age (60 days) from the stock of the Federal University of Parana were kept under specific conditions. The animals were divided by sex and kept in groups of four in standard cages for rodents (Insight, Brazil) with the following dimensions: 49 × 34 × 16 cm (length, width, and height), temperature (22 ± 2°C), humidity (50 ± 20%), and light-controlled room (12 h light/dark cycle). Filtered water and standard pellet food for rodents (Nuvital CR1, Curitiba, PR, Brazil) were offered* ad libitum*. Environmental enrichment and veterinary care were available throughout the experimental period. All experimental activities described in this study were previously approved by the Ethics Committee of the Universidade Paranaense (UNIPAR, Brazil; protocol number 20768/2011).

#### 2.3.2. Repeated Dose 90-Day Oral Toxicity Study

The doses used in this study were determined from the lowest dose (30 mg) required to pack an intermediate-size food product (30 g), and from this dose a 10 times safety factor was set (300 mg/kg), used to calculate the intermediate dose (100 mg/kg). The animals were divided into four groups of 10 rats (females and males), orally treated for 90 days with vehicle (control) or with different doses of FCSA (30, 100, and 300 mg/kg). Throughout the experimental period, the animals were daily weighed and observed for clinical signs of toxicity.

After 90 days of treatment, rats were evaluated for* “in vivo”* renal function and subsequently euthanized. Twelve hours prior to euthanasia, animals were fasted with free access to water. First, under anesthesia with isoflurane, blood was collected from the ocular plexus for hematological parameters. Subsequently, animals were decapitated (to obtain serum for evaluation of biochemical parameters) and organs were removed for determining the relative weights and histopathological analyses. All protocols used strictly followed the Guidelines for the Testing of Chemicals 408 of the Organization for Economic Cooperation and Development [9].

*(1) Clinical Observations and Measurements*. Rats were monitored throughout the study for general health/mortality and moribundity twice daily in the morning and afternoon. Cage side observations were performed on days 1–90 and detailed clinical observations, including food consumption, were performed once weekly. Body weight was measured on the day of randomization and on days 1, 8, 15, 22, 29, 36, 43, 50, 57, 64, 71, 78, 85, and 90 throughout the experimental period. Body weight was measured again at day of euthanasia to calculate the relative weight of organs.

*(2) Renal Function and Urinary Analysis*. Renal function was evaluated according to method previously described with minor modifications [10, 11]. About 24 hours before the beginning of experiment, rats were placed in individual metabolic cages (type 304 stainless steel, Tecniplast, Italy) for adaptation. After 4 hours of fasting, animals received an oral dose of 5 mL/100 g of physiological saline solution (0.9% NaCl) to provide a body uniformity of electrolytes. Urine samples were obtained every two hours for a total of eight hours. Urinary concentrations of sodium, potassium, pH, conductivity, and density were determined at the end of the experiment. In addition, urinary sediment samples were microscopically analyzed for the presence of epithelial cells, leukocytes, erythrocytes, and cylindrical and crystalline precipitates.

*(3) Hematological Evaluation*. Hematological analyses were performed on an automated analyzer with specific settings for rats (Abbott Cell Dyn 3500, Abbott Diagnostics, USA) [12]. Hematological parameters analyzed were the following: red blood cell count (106/mm^3^); total leukocyte count (103/mm^3^); platelet count (103/mm^3^); differential leukocyte count (relative number, %); determination of hemoglobin (g/dL); hematocrit (%); and determination of RBC indexes, mean cell volume (MCV, fL), mean cell hemoglobin (MCH, pg), and mean cell hemoglobin concentration (MCHC, %).

*(4) Biochemical Analyses*. Biochemical analyses used an automated analyzer (Selectra E, Vital Scientific, Netherlands). The following parameters were measured: uric acid (mg/dL), urea (mg/dL), creatinine (mg/dL), sodium (mEq/L), potassium (mEq/L), alanine aminotransferase (ALT, U/L), aspartate aminotransferase (AST, U/L), alkaline phosphatase (ALP, U/L), total serum protein (g/dL), albumin (g/dL), globulin (g/dL), total bilirubin (mg/dL), direct bilirubin (mg/dL), indirect bilirubin (mg/dL), triglycerides (mg/dL), total cholesterol (mg/dL), HDL cholesterol (mg/dL), amylase (U/L), and glucose (mg/dL).

*(5) Relative Weight of Organs*. Organs such as kidney, spleen, liver, testicles, epididymis, levator ani muscle, glans penis, seminal vesicle, prostate, and uterus of each rat/group/sex were cleaned and weighed. The average weight of paired organs was also obtained. The relative weight (%) of each organ was obtained by multiplying by 100 and divided by the body weight of each animal.

*(6) Necropsy and Pathology*. All animals underwent autopsy on all external and internal cavities. Furthermore, kidneys, spleen, brain, lungs, intestines (duodenum, jejunum, and ileum), liver, testicles, epididymis, seminal vesicles, prostate, uterus, and ovaries were carefully removed, fixed, sectioned, mounted on glass slides, and stained with hematoxylin and eosin [13]. Necropsy and histopathology evaluation were performed by a board-certified veterinary pathologist.

### 2.4. Statistical Analysis

Results are expressed as mean ± standard error of the mean of ten animals in each group. Statistical analysis was carried out using analysis of variance (ANOVA) followed by Bonferroni's test. A *p* value less than 0.05 was considered statistically significant. Graphs were drawn and statistical analysis was carried out using GraphPad Prism version 5.0 for Mac OS X (GraphPad Software, San Diego, CA, USA).

## 3. Results

### 3.1. Subchronic Toxicity of FCSA

#### 3.1.1. Clinical Measurements and Mortality

**Figure 1:**
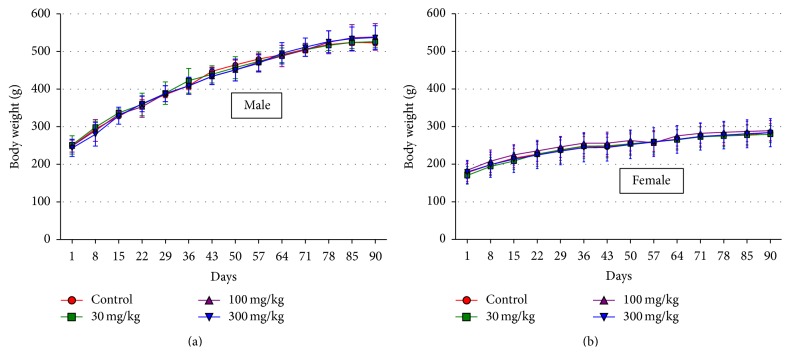
Body weights of male (a) and female (b) rats treated with distilled water (control) and FCSA (3, 30, and 300 mg/kg) for 90 days. Data are the mean ± standard deviation of the weekly body weights for each treatment group.

Signs of toxicity or deaths were not recorded throughout the 90-day study period. Weekly body weight and weekly body weight gain over the treatment period were similar among animals (both sexes) (Figures 1(a) and 1(b)). Additionally, no alterations were observed in food consumption among experimental groups.

#### 3.1.2. Effects of FCSA on Renal Function

Urine volume, pH, density, conductivity, and Na^+^ and K^+^ results for males and females rats are shown in Table 1. No changes were observed in physicochemical parameters or sediment analysis in all urine samples evaluated.

#### 3.1.3. FCSA Treatments Do Not Affect the Hematologic or Biochemical Parameters in Wistar Rats

Results for hematologic and biochemical analysis are shown in Tables 2 and 3, respectively. The results obtained indicate that values show no alterations (compared with the control group) and are within normal range to the species used in this study.

#### 3.1.4. Prolonged Treatment with FCSA Does Not Affect the Relative Weight of Organs or Induce Pathological Alterations in Wistar Rats

**Figure 2:**
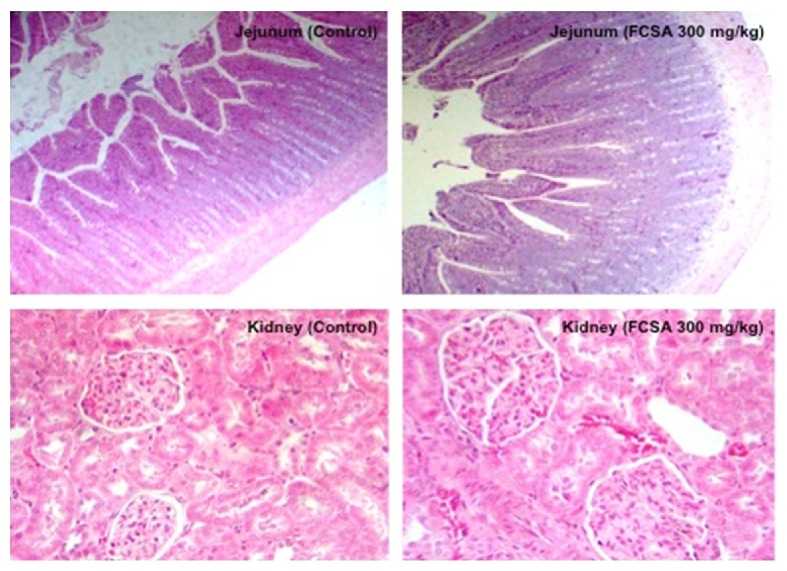
Photomicrographs of jejunum and kidney histopathology from representative male Wistar rats treated with vehicle (control group) or the highest dosage of FCSA (300 mg/kg) for 90 days. At the top, the jejunum (200x) and its respective control group are shown. In the line below, a sample of the left kidney (400x) is also represented. Hematoxylin and eosin stain.

There were no FCSA-related changes in the relative weight of organs of all experimental groups. Furthermore, no significant change was observed by autopsy or histopathological analysis in all samples. Due to alterations reported in literature, intestine and kidney samples from both male and female rats from control and FCSA groups (300 mg/kg) are shown in Figure 2. Slides show that the jejunum and kidney of high-dose rats were considered normal and had no abnormalities. All microscopic findings were of similar incidence in control and treated animals and therefore unrelated to FCSA administration.

## 4. Discussion

In recent years, a large amount of biomaterials has been developed with the aim of producing edible films used for food quality preservation [1, 3]. One of the most versatile materials with potential use in edible films is starch. It can be converted into chemicals such as acetone, ethanol, or organic acids used to produce synthetic polymers; biopolymers were produced by fermentation processes or hydrolyzed and were used as monomers or oligomers. Furthermore, chemically modified starch (such as acetylated) can be used to produce flexible films or coatings used in biodegradable packaging or as carrier for controlled drug delivery [2, 14].

Despite the potential use of starch-derived biopolymers and the belief that their natural origin ensures low toxicity, some studies have shown that even natural products commonly used as additives or coatings in food products may have toxic effects. Nevertheless, most of these studies have only investigated the toxicity of compounds obtained from microorganisms, crustaceans, or nonstarch derivatives [15–17].

For the first time, a study on the subchronic toxicology (90 days) was conducted to provide a comprehensive assessment of the risk of possible prolonged FCSA consumption. The study aimed at exploring the main parameters that are commonly affected by prolonged exposure to toxic agents, such as hematopoietic, reproductive, and nervous systems [9]. There was no disturbance in the peripheral and central nervous systems that could demonstrate abnormalities or other signs of toxicity during prolonged administration of FCSA. Moreover, FCSA treatment does not affect hematopoiesis or other hematological parameters as well as hormone responsive tissues of male or female rats. Likewise, although the observation period is relatively short, no neoplastic or nonneoplastic lesions were observed in all experimental groups.

A study conducted about 30 years ago showed that mice chronically treated with modified starch had increased water consumption and increased amounts of calcium and amorphous crystals in urine. Moreover, a slightly increased incidence of intratubular nephrosis was also observed [7]. Nevertheless, significant changes in renal function or detectable signs of histopathological lesions in animals treated with FCSA were not observed, similarly to an acute toxicity study recently conducted [18]. In fact, it is possible that methodological differences, including species, dose, and time of observation, may be involved in the apparent discrepancy found among studies.

Literature data have shown that many polysaccharides, including starch, can cause some changes in small and large intestines. Cases of changes in the cecal and colonic enlargement have been reported after use of modified starches [7]. Furthermore, it is possible that the lower metabolism of these compounds by amylase [19] could contribute to the effects described. In this context, pathological evidence in small and large intestines that could justify any change induced by FCSA in both male and female Wistar rats was not found.

Another contentious issue on starches refers to their metabolic effect on the small intestine. Diets high in carbohydrates can induce hypertriglyceridemia, hyperglycemia, and atherosclerosis as well as changes in liver function. One explanation for this fact is the inadequate evolutionary adaptation to starch and sugars in foods [20]. Considering this possibility, several markers of glucose and lipid metabolism as well as enzymes involved in the pancreatic and hepatic function were also measured, and no alterations in these clinical biochemical parameters were found.

In addition to the above data, studies have shown that different types of starch, including starch acetate, may contribute to the development of microbial population groups and short-chain fatty acids (SCFA) in the cecum and feces of rats [21]. Thus, in addition to the possible lack of toxicity presented in this work, the increased proliferation of bifidobacteria, lactobacilli, and SCFA induced by FCSA may be helpful for the suppression of pathogenic organisms in the colon. Further studies must be conducted to clarify the effects of FCSA administration on intestinal microbiota or other enzymatic systems present in the intestines.

## 5. Conclusion

Treatments with FCSA did not induce toxicity signs in all clinical parameters evaluated, including gross and microscopic pathologies. Furthermore, FCSA treatment did not affect hematopoiesis, serum biochemical parameters, or physicochemical aspects of urine after prolonged administration. Additional preclinical toxicology studies (such as mutagenicity and genotoxicity studies) and clinical trials should be carried out to complement the safety evaluation in the use of FCSA in humans, mainly due to the possibility of its use for prolonged periods of time.

## Figures and Tables

**Table 1:** Effect of FCSA on urinary parameters of male and female Wistar rats exposed for 90 days.

Parameters	Control		FCSA 3 mg/kg/day		FCSA 30 mg/kg/day		FCSA 300 mg/kg/day	
	Male	Female	Male	Female	Male	Female	Male	Female
Urine volume (mL/8 hours)	9.76 ± 1.40	10.1 ± 1.34	10.5 ± 1.78	11.2 ± 1.74	12.0 ± 1.27	9.87 ± 1.31	11.2 ± 1.43	12.1 ± 1.64
pH	6.36 ± 0.04	6.27 ± 0.13	6.48 ± 0.12	6.43 ± 0.25	6.15 ± 0.12	6.56 ± 0.15	6.18 ± 0.12	6.52 ± 0.16
Density (g/mL)	1.02 ± 0.0	1.01 ± 0.0	1.01 ± 0.0	1.01 ± 0.0	1.02 ± 0.0	1.01 ± 0.0	1.02 ± 0.0	1.01 ± 0.0
Conductivity (mS/cm)	17.6 ± 0.16	13.1 ± 1.91	17.5 ± 0.62	13.9 ± 1.31	18.0 ± 0.17	14.1 ± 1.15	18.0 ± 0.30	14.1 ± 1.47
Na^+^ (mmol/L)	113 ± 1.12	120 ± 1.47	115 ± 0.93	121 ± 1.88	116 ± 1.27	118 ± 1.86	115 ± 2.04	119 ± 1.82
K^+^ (mmol/L)	35 ± 3.08	38.7 ± 2.07	34 ± 2.25	39.8 ± 1.66	37 ± 2.06	40.9 ± 1.59	35 ± 0.89	39.4 ± 1.26

Values are expressed as mean ± S. E. M. of ten rats in each group in comparison to the control using one-way ANOVA followed by Bonferroni's test.

**Table 2:** Effect of FCSA on hematological parameters of male and female Wistar rats exposed for 90 days.

Parameters	Control		FCSA 3 mg/kg/day		FCSA 30 mg/kg/day		FCSA 300 mg/kg/day	
	Male	Female	Male	Female	Male	Female	Male	Female
RBC (10^6^/mL)	7.9 ± 0.6	7.4 ± 0.1	8.6 ± 0.1	7.3 ± 0.1	8.5 ± 0.1	7.4 ± 0.1	8.5 ± 0.2	7.5 ± 0.1
Hemoglobin (g/mL)	14.7 ± 0.4	14.4 ± 0.2	15.4 ± 0.2	14.3 ± 0.2	15.1 ± 0.1	14.4 ± 0.2	16.1 ± 0.8	14.7 ± 0.1
Hematocrit (%)	43 ± 1.2	41 ± 0.6	45 ± 0.5	40 ± 0.6	44 ± 0.4	40 ± 0.7	44 ± 1.0	41 ± 0.4
MCV (fL)	51.9 ± 0.5	56.2 ± 0.6	52.5 ± 0.5	55.7 ± 0.4	52.3 ± 0.5	55.1 ± 0.6	51.7 ± 0.3	55.8 ± 0.4
MCH (pg)	17.7 ± 0.3	19.7 ± 0.3	18.0 ± 0.2	19.6 ± 0.2	17.8 ± 0.2	19.6 ± 0.3	18.8 ± 0.7	19.7 ± 0.2
MCHC (%)	34 ± 0.3	35 ± 0.2	34 ± 0.2	35 ± 0.3	34 ± 0.2	36 ± 0.3	36 ± 1.3	35 ± 0.2
Platelets (10^3^/mm^3^)	1032 ± 79	951 ± 60	1045 ± 30	843 ± 120	1070 ± 42	881 ± 85	1098 ± 60	972 ± 71
WBC (10^3^/mm^3^)	4.4 ± 0.5	2.9 ± 0.3	5.4 ± 0.8	3.2 ± 0.8	4.4 ± 0.3	2.9 ± 0.5	4.1 ± 0.3	3.7 ± 1.3
Neutrophils (%)	24.8 ± 4.0	26.3 ± 2.4	22.2 ± 1.4	21.5 ± 3.4	21.9 ± 2.1	28.1 ± 2.9	24.5 ± 3.8	23.8 ± 1.9
Lymphocytes (%)	69.4 ± 3.4	67.3 ± 3.5	71.3 ± 2.9	71.6 ± 2.1	70.6 ± 1.7	65.5 ± 3.3	68.2 ± 2.3	69.4 ± 2.7
Monocytes (%)	4.4 ± 0.7	5.5 ± 0.7	4.9 ± 1.2	6.1 ± 1.2	5.8 ± 0.9	5.3 ± 0.8	6.1 ± 1.1	5.8 ± 1.0
Eosinophils (%)	1.4 ± 0.5	0.9 ± 0.3	1.6 ± 0.6	0.8 ± 0.2	1.7 ± 0.3	1.1 ± 0.5	1.2 ± 0.3	1.0 ± 0.3

Values are expressed as mean ± S. E. M. of ten rats in each group in comparison to the control using one-way ANOVA followed by Bonferroni's test.

**Table 3:** Effect of FCSA on biochemical parameters of male and female Wistar rats exposed for 90 days.

Parameters	Control		FCSA 3 mg/kg/day		FCSA 30 mg/kg/day		FCSA 300 mg/kg/day	
	Male	Female	Male	Female	Male	Female	Male	Female
Glucose (mg/dL)	139 ± 7.4	122 ± 5.1	139 ± 6.0	144 ± 7.1	130 ± 6.0	131 ± 5.1	137 ± 4.9	138 ± 6.2
Total cholesterol (mg/dL)	59 ± 1.4	60 ± 3.4	56 ± 2.1	57 ± 2.5	59 ± 1.7	63 ± 3.4	60 ± 2.7	63 ± 3.0
HDL cholesterol (mg/dL)	35 ± 1.1	36 ± 2.4	33 ± 1.4	33 ± 1.8	36 ± 1.1	38 ± 1.8	37 ± 1.4	39 ± 2.1
Triglycerides (mg/dL)	54 ± 6.7	56 ± 3.9	51 ± 6.7	64 ± 5.8	48 ± 5.1	54 ± 4.1	53 ± 6.5	55 ± 3.3
Urea (mg/dL)	36 ± 2.5	33 ± 3.4	38 ± 2.0	34 ± 3.4	41 ± 3.1	34 ± 2.9	44 ± 2.4	34 ± 2.8
Creatinine (mg/dL)	0.47 ± 0.02	0.48 ± 0.04	0.49 ± 0.01	0.49 ± 0.04	0.49 ± 0.01	0.50 ± 0.03	0.47 ± 0.01	0.48 ± 0.04
Sodium (mEq/L)	141 ± 1.0	138 ± 1.4	142 ± 1.3	139 ± 1.1	139 ± 0.6	137 ± 1.0	141 ± 1.3	137 ± 0.6
Potassium (mEq/L)	5.6 ± 0.1	6.0 ± 0.2	5.5 ± 0.2	6.0 ± 0.3	5.5 ± 0.1	5.8 ± 0.3	5.7 ± 0.3	5.6 ± 0.3
Uric acid (mg/dL)	1.0 ± 0.1	1.2 ± 0.1	1.2 ± 0.2	1.5 ± 0.2	1.0 ± 0.1	1.4 ± 0.1	1.1 ± 0.2	1.5 ± 0.1
Total protein (g/dL)	5.4 ± 0.1	5.4 ± 0.1	5.3 ± 0.1	5.4 ± 0.1	5.3 ± 0.1	5.5 ± 0.1	5.2 ± 0.1	5.5 ± 0.2
Albumin (g/dL)	3.5 ± 0.1	3.7 ± 0.1	3.5 ± 0.1	3.7 ± 0.1	3.5 ± 0.1	3.8 ± 0.1	3.4 ± 0.1	3.8 ± 0.1
Globulin (g/dL)	1.9 ± 0.1	1.7 ± 0.1	1.8 ± 0.1	1.7 ± 0.1	1.8 ± 0.1	1.7 ± 0.1	1.8 ± 0.1	1.7 ± 0.1
Amylase (U/L)	732 ± 24	722 ± 76	699 ± 41	667 ± 58	703 ± 37	664 ± 67	756 ± 41	596 ± 59
Alkaline phosphatase (U/L)	154 ± 11	106 ± 9	154 ± 11	107 ± 15	139 ± 9	106 ± 4	153 ± 11	100 ± 7
AST (U/L)	99 ± 9	124 ± 6	120 ± 9	117 ± 7	98 ± 8	117 ± 6	99 ± 10	134 ± 12
ALT (U/L)	49 ± 2	42 ± 2	58 ± 6	40 ± 2	50 ± 1	42 ± 2	53 ± 5	41 ± 2
Total bilirubin (mg/dL)	0.15 ± 0.01	0.12 ± 0.01	0.17 ± 0.02	0.13 ± 0.01	0.16 ± 0.01	0.19 ± 0.04	0.15 ± 0.01	0.15 ± 0.01
Direct bilirubin (mg/dL)	0.10 ± 0.01	0.07 ± 0.01	0.11 ± 0.02	0.08 ± 0.01	0.11 ± 0.01	0.10 ± 0.02	0.09 ± 0.02	0.10 ± 0.01
Indirect bilirubin (mg/dL)	0.05 ± 0.01	0.05 ± 0.01	0.06 ± 0.01	0.05 ± 0.01	0.05 ± 0.01	0.09 ± 0.01	0.06 ± 0.02	0.05 ± 0.01

Values are expressed as mean ± S. E. M. of ten rats in each group in comparison to the control using one-way ANOVA followed by Bonferroni's test.
